# Going with the membrane flow: the impact of polarized secretion on bulk plasma membrane flows

**DOI:** 10.1111/febs.16287

**Published:** 2021-12-06

**Authors:** Veneta Gerganova, Sophie G. Martin

**Affiliations:** ^1^ Department of Fundamental Microbiology University of Lausanne Switzerland; ^2^ Present address: Niels Bohr Institute University of Copenhagen Blegdamsvej 17 2100 Copenhagen

**Keywords:** cell polarity, endocytosis, exocytosis, membrane, patterning, secretion, self‐organization

## Abstract

Even the simplest cells show a remarkable degree of intracellular patterning. Like developing multicellular organisms, single cells break symmetry to establish polarity axes, pattern their cortex and interior, and undergo morphogenesis to acquire sometimes complex shapes. Symmetry‐breaking and molecular patterns can be established through coupling of negative and positive feedback reactions in biochemical reaction‐diffusion systems. Physical forces, perhaps best studied in the contraction of the metazoan acto‐myosin cortex, which induces cortical and cytoplasmic flows, also serve to pattern‐associated components. A less investigated physical perturbation is the in‐plane flow of plasma membrane material caused by membrane trafficking. In this review, we discuss how bulk membrane flows can be generated at sites of active polarized secretion and growth, how they affect the distribution of membrane‐associated proteins, and how they may be harnessed for patterning and directional movement in cells across the tree of life.

## Graphical abstract

Sites of polarized secretion induce lateral bulk plasma membrane flows. Peripheral proteins with fast unbinding and lateral diffusion dynamics can repopulate secretion sites. By contrast, peripheral proteins with long‐enough membrane residence time couple to these flows and are displaced from secretion zones. This phenomenon can be harnessed by the cell for self‐organization.

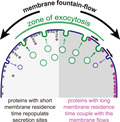

**Abbreviations**
CIBN: N‐terminus of CIB1 protein
CRY2: cryptochrome 2

## Introduction

The plasma membrane, which delimits the cell from its environment, is a highly specialized organelle where functional domains are formed through the spatial organization of lipids and proteins. Although membrane domains may appear stable through time, plasma membrane components continually turn over. In eukaryotic cells, a major form of turnover takes place through endo‐ and exocytic routes that retrieve and deliver vesicles from and to the plasma membrane, respectively. Many cell types exhibit highly polarized zones of secretion, where new membrane is added from internal pools at high rate. Examples include the tip of polar‐growing cells like fungi or pollen tubes, where cell wall remodeling enzymes are secreted; B and T cell immunological synapses, where cytolytic enzymes are released; the leading edge of migrating cells and growth cones, where adhesion molecules must be delivered; neuronal synapses, where secretion of information‐containing neurotransmitters takes place or the apical domain of exocrine cells. Because the composition of secretory vesicle membranes is not identical to that of the plasma membrane, the massive incorporation of secretory vesicles is a perturbing factor for plasma membrane homeostasis. How does the cell deal with this incorporation and what effect does the process have on the composition and spatial organization of the plasma membrane?

In this viewpoint article, we discuss how polarized secretion triggers bulk plasma membrane flows away from the secretion zone. We first present our recent findings in the simple fission yeast, where polarized secretion causes in‐plane bulk membrane flows away from the secretion site, with important consequences on the distribution of membrane‐associated proteins and cell morphogenesis. We then discuss conditions necessary to generate membrane flows, the effect flows have on membrane‐associated proteins and how this phenomenon may be used in cells to generate functional patterns.

## Observations of bulk membrane flows

**Fig. 1:**
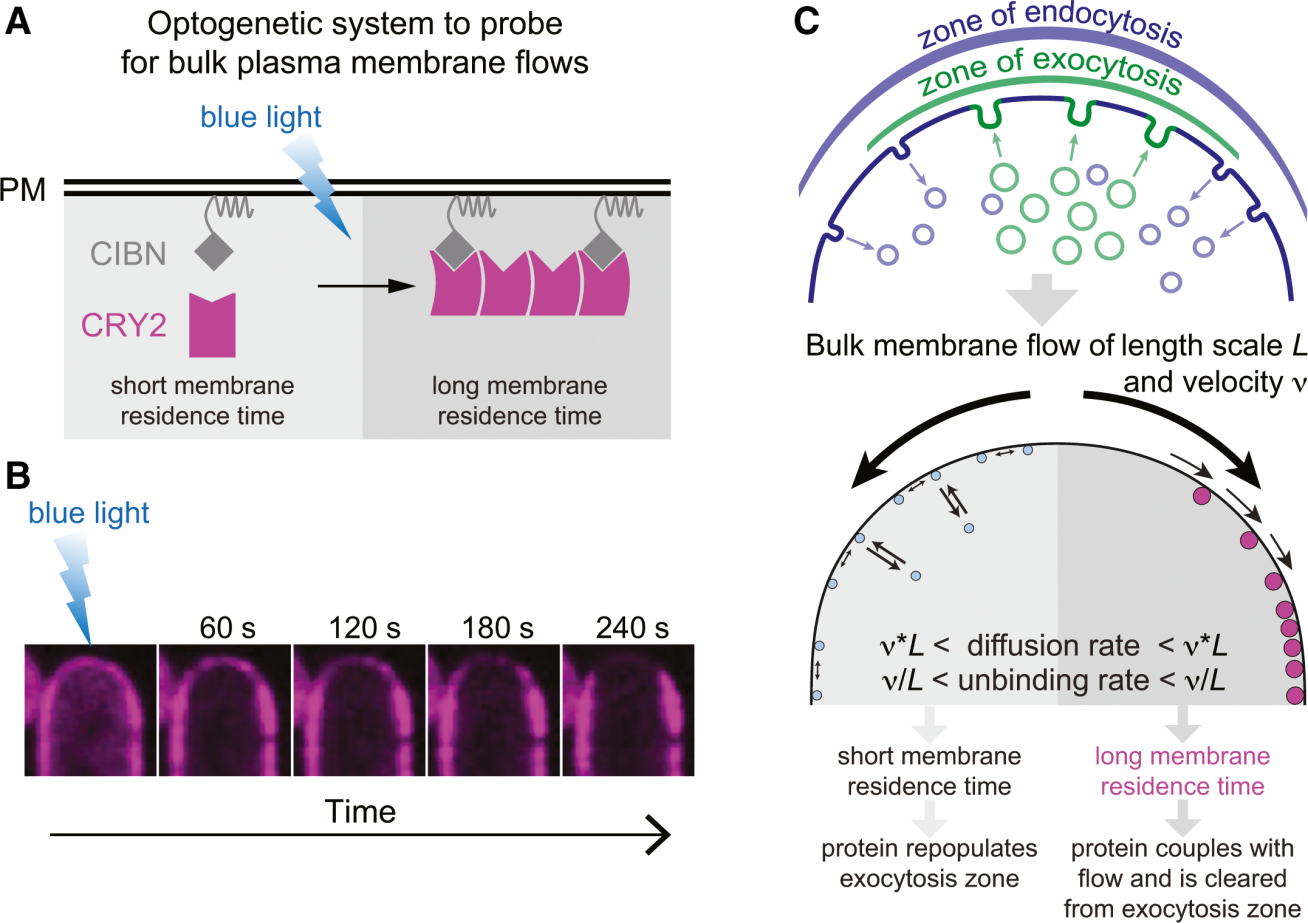
In‐plane bulk plasma membrane flows and how to detect them. (A) Schematic representation of the CRY2‐CIBN optogenetic system to probe for bulk plasma membrane flows. CIBN is a linked to the plasma membrane by the RitC peptide, which binds peripherally and exchanges rapidly with the cytosolic pool. Blue light triggers CRY2‐CIBN and CRY2–CRY2 binding, leading to the formation of oligomers with longer membrane residence time. (B) Localization of CRY2‐mCherry in *S*. *pombe* immediately after illumination, when it decorates the membrane homogeneously, and over the next 240 s showing depletion from the growing cell pole. (C) Schematic representation of membrane flows, generated by the distinctive distribution of endo‐ and exocytic events. Low affinity membrane bound proteins can occupy the zone of active growth and secretion, while higher membrane affinity proteins are pushed along by the membrane flows away from the secretion zone.

The fission yeast *Schizosaccharomyces pombe* grows by tip extension forming rod‐shaped, walled cells. The cell poles and medial division site are regions of new membrane and cell wall material delivery by polarized secretion. Using the CRY2‐CIBN optogenetic system, we recently revealed bulk membrane flows from secretion sites [1]. In our setup, the CIBN ligand associates with the plasma membrane peripherally by linkage with a small binding peptide (Fig. 1A). In the dark, CIBN rapidly exchanges between membrane and cytosol, exhibiting a homogeneous distribution at the membrane, while the light‐sensitive CRY2 is present in the cytosol. Upon light exposure, which triggers both CRY2‐CIBN and CRY2‐CRY2 binding, CRY2 is initially recruited homogeneously to its binding partner at the membrane. However, the oligomerized CRY2‐CIBN complex, which now displays increased membrane affinity, is rapidly depleted from regions of polarized exocytosis (Fig. 1B), with clusters moving away from sites of membrane insertion. These observations are best explained in simulations and supporting experiments by a fountain‐flow mechanism, where ‘naked’ membrane addition at the secretion site and retrieval by endocytosis over a broader zone causes in‐plane membrane flows away from the insertion site. Membrane‐associated proteins with sufficiently long residence time at the plasma membrane, which is the case for the oligomerized CRY2‐CIBN complex but not CIBN alone, couple to the flow and become displaced from sites of polarized exocytosis (Fig. 1C). As discussed below, this phenomenon is harnessed by the cell for morphogenesis.

## Conditions to generate bulk membrane flows

**Fig. 2:**
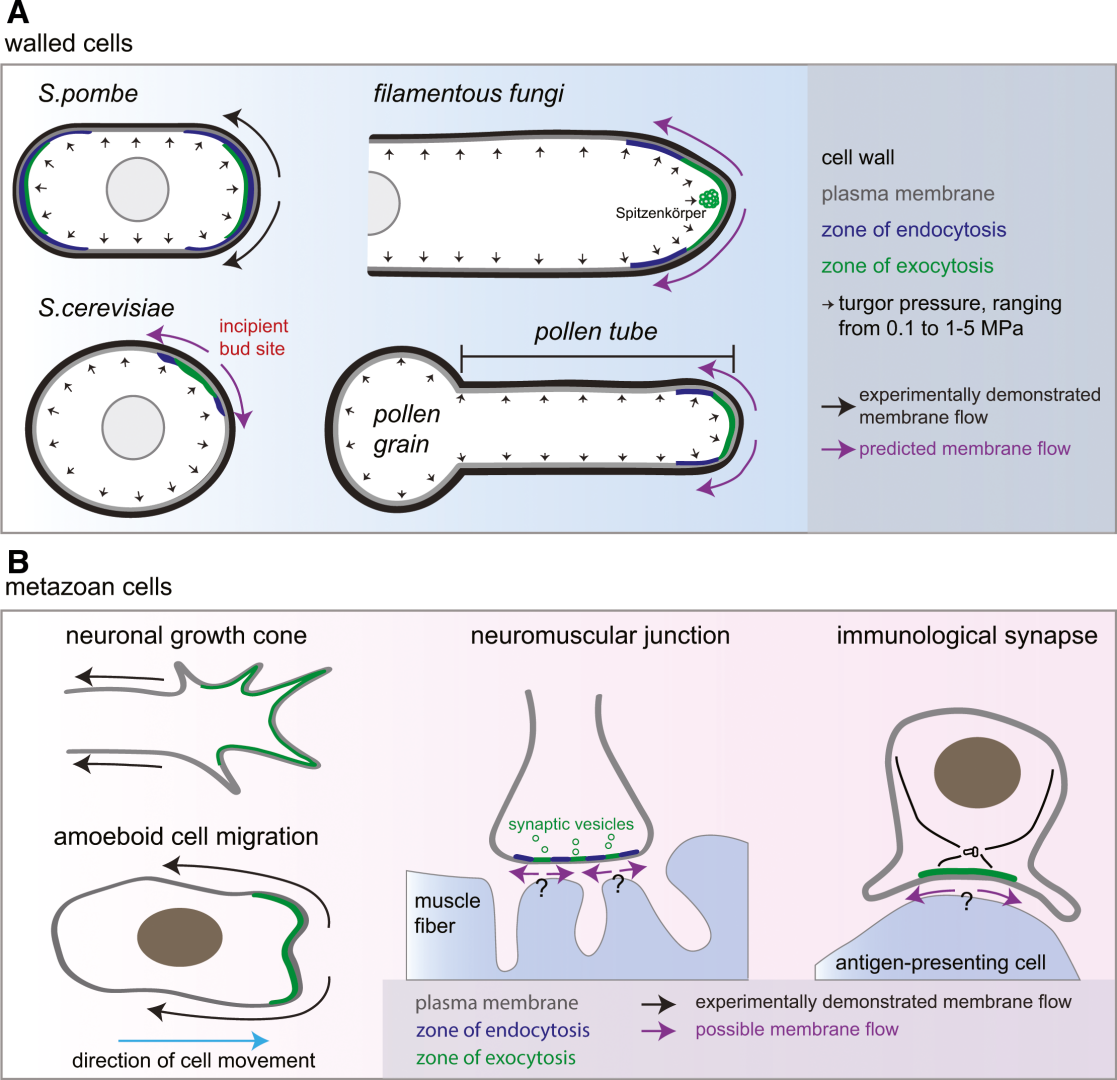
Cells in which bulk membrane flows may form. Schematic representation of the organization of endo and exocytotic events and resulting or predicted membrane flows in (A) cell‐walled organisms and (B) mammalian cells. Mammalian cells typically exhibit an actomyosin cortex (not shown), which modulates tension equilibration in the plasma membrane and thus modifies the distance at which flows may propagate.

Membranes can be considered as 2D incompressible fluids. Thus, new membrane addition will lead to expansion of the membrane surface area. Whether a secretion site generates bulk membrane flows depends on two general conditions. First, the plasma membrane must be under an overall background in‐plane tension such that new membrane addition does not lead to formation of a fold but to displacement of the pre‐existing membrane away from the site of insertion. Second, to create directional flow under homeostatic conditions, retrieval of excess membrane by endocytosis must occur in a region wider or distinct from the site of membrane insertion, a setup prevalent across cell types (Fig. 2).

Let us first examine these conditions in walled cells, which are ubiquitous in the fungal and plant phyla. These types of cells simplify the examination of membrane flows because (a) the plasma membrane is under significant tension – in electron micrographs, it generally appears as a flat surface pressed against the cell wall by the strong internal turgor pressure; and (b) walled cells do not harbor an actomyosin cortex. Tip‐growing walled cells (for instance many fungi and plant cells such as pollen tubes or root hairs) exhibit highly polarized secretion at their growing pole(s), which leads to a substantial excess of membrane exocytosis relative to the amount of new membrane required for cell growth, perhaps required to deliver sufficient cell wall remodeling material. In fission yeast cells, we and others estimated a 75–80% excess [1, 2], with similar estimations in budding yeast [3]. Excess of at least 12.5% has also been estimated in the filamentous fungus *Neurospora crassa* [4]. Similarly, there may be about 80% excess membrane delivery in pollen tubes and plant root hairs [5]. The excess membrane is recycled by endocytosis, typically over an area wider than the zone of secretion. In *S*. *pombe*, endocytosis occurs at zones of polarized secretion, but over a wider region [1, 6]; in *S*. *cerevisiae*, endocytosis forms a ring around the secretion site at the time of bud emergence [7, 8]; in filamentous fungi, the zone of endocytosis is largely distinct from the secretion zone, forming a collar around the exocytic apical zone (reviewed in [9]). Similarly, exo‐ and endocytosis sites overlap at the apex of pollen tubes [10], but these display a clear sub‐apical region where endocytosis takes place [11, 12], suggesting an organization very similar to that observed in filamentous fungi. In this configuration, computational simulations showed that membrane flows would occur even without net membrane growth [1]. The strongly polarized secretion and wider zone of membrane retrieval predicts that membrane flows are widely present in tip‐growing fungal and plant cells (Fig. 2A).

Large‐scale exocytic events are a hallmark for a number of specialized metazoan cells. Examples include neuronal growth cones and synapses, immunological synapses or exocrine cells. There is clear evidence of endocytosis‐exocytosis spatial coupling at the neuronal synapse where exocytic vesicles localize to active zones surrounded by endocytic vesicles [13] (Fig. 2B). In *Drosophila* neuromuscular junctions, the endocytic machinery surrounds sites of secretion for synaptic vesicle recycling [14], at setup compatible with the presence of flows. Exocytosis preferentially occurs at neuronal growth cones [15] and front‐directed polarized membrane trafficking may be a general feature of migrating cells [16]. Though zones of exo‐ and endocytosis have not been systematically compared in these cells, membrane flows have been described in some cell types (discussed in the next section).

Targeted vesicular trafficking is also critical for the process of cell division in all organisms [17]. In yeast and large animal cells, such as oocytes, exocytosis takes place at the leading edge as well as further back along the ingressing furrow. In animal cells, exocytosis is essential for the late steps of cell division [18]. In fission yeast, secretory vesicles are targeted along the cleavage furrow and at the rim of the division plane through distinct tethering complexes [19]. The location of endocytosis has not been precisely mapped, but, at least in yeast, the region is wider than that of exocytosis [1]. In these cells, we observed that membrane‐associated proteins deplete from the division site and display long‐range retrograde movements, suggesting long‐range bulk flows. Curiously, previous work also proposed membrane flows upon RhoA‐induced furrow formation in animal cells, but towards the division furrow [20].

While distinct distribution of exo‐ and endocytic activities may drive in‐plane membrane flows (depending on membrane tension, discussed below), cells can also resolve the destabilization caused by large‐scale membrane insertion in other ways. Solutions to this problem include kiss‐and‐run mechanisms in neurons, where the vesicle only briefly fuses with the plasma membrane to release its content but reseals before membrane integration (reviewed in [13]), or vesicle crumpling in exocrine cells, where the content of large vesicles is squeezed out of the cell before targeted vesicle membrane retrieval by endocytosis [21]. Incomplete fusion events are also reported at the immunological synapse of natural killer cells to decrease the need for extensive membrane recycling [22].

## Effect of membrane flows on mammalian cell migration

A hypothesis of bulk retrograde membrane flows driving metazoan cell migration was first formulated by [23]. In this opinion piece, Bretscher argued that membrane flowing from front to back, powered by front‐polarized exocytosis and dispersed endocytosis, drives cell migration. He also hypothesized that lateral diffusion of individual proteins would homogenize membrane composition against the flow, but that reduction in diffusion rates through protein aggregation (for instance driven by surface antigen–antibody binding), would lead to accumulation at the back of the cell. These hypotheses are supported by recent work showing that retrograde membrane flows, induced by polarized membrane trafficking and coupled to retrograde acto‐myosin contractility, drive amoeboid cell movement (independently of surface adhesion) (Fig. 2B) [24]. In these cells, surface‐bound fluorescent beads were shown to travel backwards from the leading cell edge, indicative of rearward flow. Membrane fountain‐flow linked to polarized trafficking was also observed in migrating apicomplexan cells [25]. In neurons, retrograde membrane flow, which has a significantly faster rate than cell protrusion and is dependent on membrane trafficking, similarly occurs from the axon growth cone to the cell body [26].

However, the notion of such a ‘fluid drive’ is not universal [27], for two main reasons. First, membrane flows were not observed in cells migrating on a surface through lamellipodial extension [28, 29, 30], where migration relies on surface adhesion and pushing forces generated by the actin cytoskeleton at the cell front. Second, tension in mammalian cell membranes was shown not to equilibrate within experimental timescales [31]. Instead, equilibration is likely limited by connections between transmembrane proteins and the underlying actomyosin cortex, resulting in a gel‐like membrane [32]. Absence of tension propagation likely underlies the need to coordinate exocytosis at the front and endocytosis at the back of the cell to supply membrane for cell movement. By contrast, tension equilibration was observed in cellular blebs disconnected from the cortex [31]. These observations are backed‐up by observation of motions of transmembrane protein tracers, showing strong correlation at short length scales (< 1 µm), but no correlation at longer length scales, indicating membrane flow arrest [33]. Thus, the distance over which tension propagates likely varies in different cell types and cellular regions, which sets the length scale at which bulk membrane flows may be observed.

## Effect of membrane flows on the distribution of membrane‐associated proteins

What is the effect of membrane flows on the distribution of membrane‐associated proteins? In general, flows are predicted to influence the steady‐state distribution of proteins, depending on their residence time at the plasma membrane and on their relative abundance in vesicles and the plasma membrane [1].

In the simplest situation, the protein associates specifically with the plasma membrane but does not bind transport vesicles, such that secretion indeed adds ‘naked’ membrane. This is the case of many proteins that associate peripherally with the plasma membrane, for instance through amphipathic helices or other lipid‐binding domains. Such proteins do not depend on vesicular trafficking but dynamically exchange between cytosol and membrane, with a steady‐state distribution that depends on membrane‐binding and unbinding rates. Proteins that exhibit fast unbinding rates can populate the ‘naked’ membrane at the secretion site fast enough to display a homogenous distribution irrespective of bulk membrane flows. By contrast, proteins with a long membrane residence time, due to slow unbinding rates or slow lateral mobility, couple with membrane flows and become displaced from sites of new membrane addition. Indeed, increasing membrane residence time through simple tandem dimerization of a membrane‐binding domain is sufficient to convert a homogeneous localization to one depleted from secretion sites [1]. Quantitatively, for flows with velocity *v* over length scale *L*, proteins that exhibit diffusion coefficients smaller than *v*·*L* and detachment rates smaller than *v*/*L* will be depleted from the secretion site [1] (Fig. 1C). Although peripheral membrane proteins typically exhibit relatively fast diffusion rates, membrane unbinding and lateral diffusion rates may be modulated by post‐translational modification, tandem linkage or oligomerization [34, 35], slowing them to a range compatible with flow coupling. Indeed, light‐induced oligomerization of CRY2, leading to longer membrane residence time, causes the depletion we observed at the poles of the fission yeast cell [1]. Thus, bulk membrane flows modulate the steady‐state distribution of slow peripheral membrane‐associated proteins.

The situation is more complex for proteins that also associate with vesicles, as is the case of transmembrane proteins, which are delivered to the plasma membrane by secretion. As above, bulk membrane flows are predicted to influence their steady‐state distribution only if they have slow enough lateral mobility, which prior work showed is modulated by protein densities at the membrane [36]. While transmembrane proteins typically have slower lateral diffusion rates compared to peripheral proteins and may thus couple to flows leading to local depletion, their delivery by secretory vesicles may compensate this depletion. Endocytosis also plays an important role to ‘detach’ these proteins from the plasma membrane. Their steady‐state localization will thus depend on their relative abundance in secretory vesicles and at the plasma membrane, as well as on their inclusion or exclusion from endocytic vesicles. This leads to distributions that can range from depletion at secretion sites, in the case of lower concentration in the secretory vesicle membrane than at the plasma membrane, to uniform distribution, to even strong enrichment at the secretion site upon efficient retrieval by endocytosis in neighboring regions [37].

Does bulk flow also affect lipid distribution? As the membrane lipid composition changes drastically across the secretory system, with for instance the plasma membrane displaying the highest level of sterol and sphingolipids [38], secretory vesicle delivery is likely to locally alter the composition of the target plasma membrane. However, lipids exhibit a strong lateral mobility with lateral diffusion rates in the range of 1 to 20 µm^2^·s^−1^ [39], which allows for rapid mixing. Therefore, the newly added membrane may acquire an identical lipid composition to the surrounding plasma membrane almost instantaneously. Lipid microdomains may alter this behavior displaying reduced collective diffusion distinct from that of individual lipids coming in and out.

Individual molecules may display distinct behavior relative to the bulk of the membrane, with components being immobile for instance through interaction with static cellular structures [40]. In yeast cells, transmembrane proteins are largely immobile, perhaps due to interaction with the cell wall, and so are unlikely to be strongly affected by membrane flows. However, collectively, molecules associated long enough with the fluid component of the membrane will flow with it.

## Consequences of membrane flows on cell organization

What is the consequence of membrane flows on cell patterning? Because secretion sites cause bulk membrane flows, membrane‐associated proteins may be differentially localized depending on their membrane‐binding properties, as discussed above. In fission yeast, we showed that membrane flows participate in a negative feedback loop to shape the cell. The rod shape is achieved by cortical zones of active Cdc42 GTPase promoting polarized secretion of cell‐wall remodeling enzymes. The size of the active zones defines cell width. The zone width is itself restricted by Cdc42 GTPase activating proteins (GAP) that localize to cell sides and inhibit Cdc42, including the GAP Rga4, whose localization is likely patterned by membrane flows [1]. By constructing an artificial Cdc42 GAP, whose oligomerization state and thereby membrane residence time, is acutely modified by light, we engineered cells that changed their shape in response to light [1]. In the dark, the GAP localized uniformly, inhibiting Cdc42 function and yielding round cells; in the light, it coupled to membrane flows, thus allowing zones of Cdc42 activity and establishing the rod shape. Therefore, in fission yeast cells, bulk membrane flows are an intrinsic mechanism of cell morphogenesis.

There is an intriguing parallel between Cdc42 GTPase regulation by the Rga4 GAP in fission yeast and ROP1 GTPase regulation by the recently described REN4 protein in *Arabidopsis* pollen tubes. REN4 functions as a negative regulator of ROP1, by promoting its endocytic retrieval in the sub‐apical zone, thus limiting the active ROP1 zone. Interestingly, REN4 associates with the apex of non‐growing pollen tubes but is displaced laterally in growing tubes [41]. It will be interesting to probe whether its localization, like that of Rga4, is modulated by bulk flows.

We hypothesize that many protein localizations and morphogenetic events are shaped by membrane flows. These may include the sculpting of the septin ring by exocytosis and establishment of daughter cell identity in *S*. *cerevisiae* [42], or the exclusion from cell poles of a number of proteins determining fission yeast cell shape and division [1]. An interesting, as‐yet untested idea is that the oft‐observed distribution of endocytosis around sites of exocytosis is a consequence of membrane flows. How the two activities are coupled is still poorly understood, but at least two mechanisms have been proposed. In *S*. *cerevisiae,* the Rab GTPase Sec4, which promotes the tethering of secretory vesicles with the plasma membrane, also activates actin polymerization for endocytosis, thus promoting endocytic events in the vicinity of exocytic sites [43]. In the same organism, cargo‐binding was recently proposed to drive the maturation and internalization of endocytic sites [44], with similar evidence reported in mammalian cells [45]. As many cargoes are recycling proteins recently delivered by secretory vesicles (such as v‐SNAREs), this provides another spatial coupling mechanism. Displacement of such cargoes by secretion‐induced flow would be predicted to yield a peripheral ring of endocytosis around sites of secretion, as observed in filamentous fungi and pollen tubes. Looking forward, it will be exciting to test where membrane flows occur in cells – this can in principle be done by using the optogenetic CIBN‐CRY2 system described above and comparing the distribution of CIBN in dark and light states – and probe how generally membrane flows contribute to cellular patterning.

## Conflict of interest

The authors declare no conflict of interest.

## Author contributions

VG and SGM co‐wrote the manuscript and designed the figures.

## Data availability statement

All data is available within the article.
